# Supplementary material for: Ability of Delta Radiomics to Predict a Complete Pathological Response in Patients with Loco-Regional Rectal Cancer Addressed to Neoadjuvant Chemo-Radiation and Surgery
Source: Cancers (Basel). 2022 Jun 18;14(12):3004. doi: 10.3390/cancers14123004 (PMC9221458; doi:10.3390/cancers14123004)
Supplement: Supplementary file 1 [file cancers-14-03004-s001.zip › cancers-1644157-supplementary.pdf]

## SUPPLEMENTARY MATERIALS

**Table S1.** Texture analysis parameters calculated with Lifex Software, and corresponding description.

| Type of radiomics Feature | Radiomics Feature Name | Description |
|---|---|---|
| **Indices from Histogram:** provides informations derived from global histogram analysis | Volume.ml | Measures the volume in voxels or millilitre |
| | Skewness | measures the asymmetry of the gray-level distribution in the histogram. |
| | Kurtosis | measures whether the gray-level distribution is peaked or flat relative to a normal distribution. |
| | Entropy | mesures the randomness of the distribution |
| | Energy | measures the uniformity of the distribution |
| **Indices from Sphericity** | Sphericity | Measures how spherical a Volume of Interest is |
| | Compacity | Measures the degree to which the Volume of Interest is compact |
| **Co-occurrence Matrix (GLCM)**: takes into account the arrangements of pairs of voxels to extract textural indices. | GLCM.homogeneity | Homogeneity of gray-level voxel pairs |
| | GLCM.energy | Uniformity of gray-level voxel pairs. |
| | GLCM.contrast | Local variations in the GLCM. |
| | GLCM.correlation | Linear dependency of gray-levels in GLCM. |
| | GLCM.entropy | Randomness of gray-level voxel pairs. |
| | GLCM.dissimilarity | Variation of gray-level voxel pairs. |

**Table S2.** Reliability analysis of TA parameters.

| TA Parameter | T2-MRI ICC (single measure) | DWI-MRI ICC (single measure) | ADC-MRI ICC (single measure) |
|---|---|---|---|
| Volume.ml | 0.970 | 0.887 | 0.881 |
| Skewness | 0.743 | 0.912 | 0.826 |
| Kurtosis | 0.387 | 0.925 | 0.943 |
| Entropy | 0.588 | 0.735 | 0.873 |
| Energy | 0.584 | 0.356 | 0.825 |
| Sphericity | 0.847 | 0.393 | 0.868 |
| Compacity | 0.711 | 0.772 | 0.756 |
| GLCM.homogeneity | 0.757 | 0.726 | 0.806 |
| GLCM.energy | 0.688 | 0.234 | 0.590 |
| GLCM.contrast | 0.238 | 0.807 | 0.727 |
| GLCM.correlation | 0.656 | 0.846 | 0.431 |
| GLCM.entropy | 0.811 | 0.736 | 0.866 |

| | | | |
|---|---|---|---|
| GLCM.dissimilarity | 0.715 | 0.838 | 0.745 |

**Table S3.** Logistic regression analysis performed on both Training Dataset and Validation Dataset.

| Logistic Regression Analysis | | | | | |
|---|---|---|---|---|---|
| **Endpoint** | **Parameter** | **p-value** | **B** | **OR (95% CI)** | **Dataset** |
| **TRG 1** | ADC GLCM Delta Entropy | <0.001 | -1.97 | 0.14 (0.03-0.54) | Training dataset |
| **TRG 1** | ADC GLCM Delta Entropy | 0.001 | -2.48 | 0.08 (0.01-0.73) | Validation Dataset 1 |
| **TRG 1** | ADC GLCM Delta Entropy | 0.008 | -3.43 | 0.06 (0.01-0.052) | Validation Dataset 2 |

**Table S4.** Characteristics of the ROC Curves. 2LL: 2 log-likelihood; $R^2$: Nagelkerke $R^{2,}$, AUC: Area Under the Curve of the ROC; SE: standard error; HL: Hosmer–Lemeshow.

| | **Training Dataset** | **Validation Dataset 1** | **Validation Dataset 2** |
|---|---|---|---|
| **-2LL** | 28.46 | 19.01 | 17.23 |
| **$R^2$** | 0.477 | 0.507 | 0.469 |
| **AUC** | 0.874 | 0.926 | 0.888 |
| **SE** | 0.058 | 0.048 | 0.089 |
| **HL $X^2$** | 3.92 | 4.66 | 8.31 |
| **HL *p*-value** | 0.77 | 0.79 | 0.40 |
